# Ultrasound Elastography Use in Lower Extremity Lymphedema: A Systematic Review of the Literature

**DOI:** 10.7759/cureus.5578

**Published:** 2019-09-05

**Authors:** Antonio J Forte, Maria T Huayllani, Daniel Boczar, Gabriela Cinotto, Sarah A McLaughlin

**Affiliations:** 1 Plastic Surgery, Mayo Clinic Florida - Robert D. and Patricia E. Kern Center for the Science of Health Care Delivery, Jacksonville, USA; 2 Surgery, Mayo Clinic Florida - Robert D. and Patricia E. Kern Center for the Science of Health Care Delivery, Jacksonville, USA

**Keywords:** lymphedema, lower extremity, ultrasound elastography

## Abstract

Lower extremity lymphedema (LEL) is mainly assessed clinically. Ultrasound elastography (UE) is a promising imaging tool to assess this disorder. We conducted a systematic literature review to describe the studies evaluating the use of UE in LEL. The PubMed database was queried for studies that evaluated the use of UE in LEL. The keywords “elastography” AND “lymphedema” were used for the search. Original articles in English were included in our study, whereas reviews were excluded. Our search resulted in 12 articles, 4 of which met the inclusion criteria. UE methods included free-hand real-time tissue elastography and UE with transducer in B mode. The imaging parameters applied were the tissue strains and the area of red region, respectively. All studies tested UE use in the assessment of LEL, and only one considered its use for staging. All studies but one found a difference in strain parameters for assessment of patients with LEL. Our systematic review has shown that UE appears to be a great tool in the assessment of LEL in moderate-to-advanced stages of disease. However, further studies using new effective methods are needed to evaluate patients with early lymphedema.

## Introduction and background

Lower extremity lymphedema (LEL) is a chronic condition characterized by an extravasation of fluid into the interstitial spaces [1]. It is usually secondary to surgery or radiotherapy for the treatment of malignancies, such as genitourinary (penile, prostate, bladder, penile) and gynecologic (ovarian, cervical, endometrial, vulvar) malignancies and lower limb melanomas [2,3]. Incidence of secondary LEL accounts for 20% of the reported cases [3].

Diagnosis of lymphedema is principally clinical, with characteristics including swelling, pitting to non-pitting edema progression, slow reduction of swelling with elevation of lower affected limbs, dorsal hump of the foot, Kaposi-Stemmer sign (inability to pinch the fold of skin on the dorsal area of the base of the second toe), and square toes [4]. These signs are often better visualized as the disease progresses, with a sensitivity of 17%, specificity of 88%, and an overall accuracy of 47% in predicting lymphoscintigraphy-confirmed lymphedema for all the mentioned signs [5]. This low percentage underscores the importance of specific tools for prompt diagnosis and assessment of patients with LEL. Diagnostic tests like lymphoscintigraphy, lymphangiography, magnetic resonance, computed tomography lymphangiography, and ultrasound are useful for the diagnosis and assessment of these patients [6]. However, high costs, invasiveness, radiation dosage issues, lack of diagnostic parameters, and unreliable results have limited their standard use of most of them [5]. Ultrasound is of particular interest due to its advantages of low cost and easy availability [7]. Specifically, ultrasound elastography (UE) is an imaging tool that assesses tissue stiffness to differentiate affected tissue from normal tissue. Its use has been studied in many solid breast, thyroid, kidney, and prostate tumors, as well as in lymph nodes and chronic liver diseases [7], but not so well for the diagnosis and assessment of patients with LEL.

In this review, we provide an overview of the studies that have applied UE in patients with LEL, the UE method, and possible parameters, as well as discuss the results, application, and limitations of its use.

## Review

Materials and Methods

Study Selection

This systematic review included original articles of studies evaluating the use of UE in patients with LEL that were written in English. Reviews were excluded, as were articles in which use of UE was not assessed in patients with LEL.

Data sources and search strategy

Guidelines outlined in the Preferred Reporting Items for Systematic reviews and Meta-Analyses (PRISMA) were followed. A comprehensive systematic review was conducted by one author (M.T.H.) on June 25, 2019, using the PubMed database, and querying for articles reporting the use of UE in LEL. The keywords for the search strategy were “elastography” AND “lymphedema”. 

Studies were identified and uploaded into EndNote (Clarivate). Manuscripts were screened manually and selected according to the inclusion and exclusion criteria in a two-step process by two authors (M.T.H., D.B.). First, studies were reviewed based on the title and abstract. Then, the full texts of the selected manuscripts were screened. If one author doubted selecting an article, the other author reviewed the article according to the selection criteria and both reviewers came to a consensus. 

Data pooling and data analysis

Relevant data were extracted and pooled describing the author, year, participants, age of participants, International Society of Lymphology (ISL) stage, type of ultrasound, method, imaging biomarker used to measure the results, standard comparison tool, reason for study and outcomes.

Results

**Figure 1:**
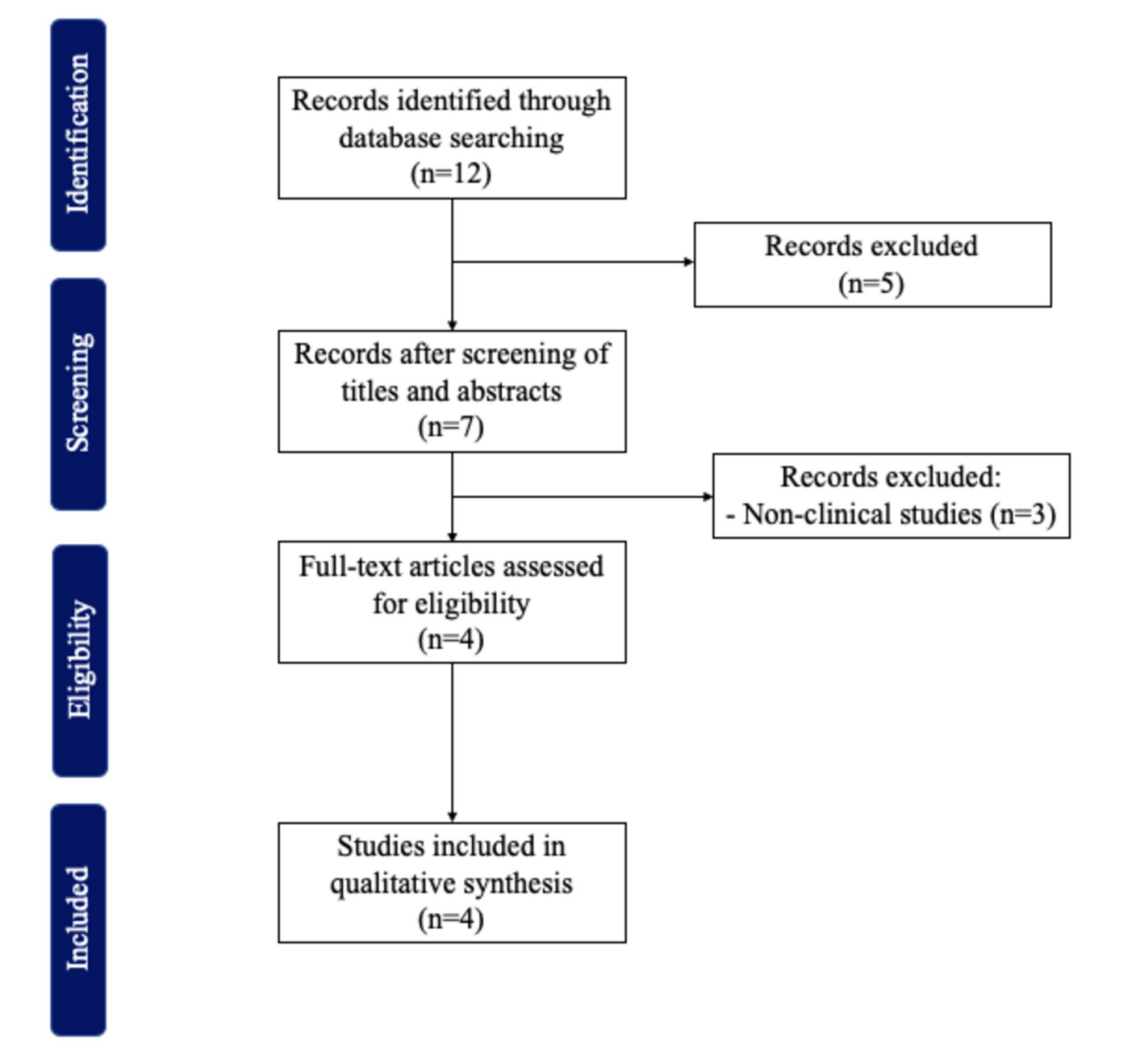
Inclusion and exclusion criteria

Of the 12 articles found, 4 met the inclusion criteria (Figure 1). All included studies were published between 2014 and 2016. Detailed descriptions of these studies are provided in Table 1. UE methods tested included elastography with transducer in B mode and free-hand real-time elastography (RTE) [8-11]. Imaging biomarkers used to compare the efficacy of the tested UE method were skin and subcutaneous tissue strains and area of red region in the tissue [8-11]. All articles reported the use of UE for assessment of patients with lymphedema, and one also included its use in staging of the disease [12]. All, but one of the studies found differences between the limb affected by lymphedema and the non-affected limb [11].

**Table 1:** Studies Analyzing the Use of Ultrasound Elastography in Lower Extremity Lymphedema Abbreviations: RTE, real time tissue elastography; ISL, International Society of Lymphology; LDS, lipodermatosclerosis; MLD, manual lymph drainage; ICG, indocyanine green

Author	Year	Participants	Age	ISL Stage	Type of Ultrasound	Method	Imaging Biomarker	Standard comparison tool	Reason for Study	Outcomes
Suehiro et al [9]	2016	18 patients (20 legs with secondary lymphedema)	Median (range): 65 (37-84)	Stage II (n=18), stage III (n=2)	Ultrasound system (HI VISION Preirus, Hitachi Aloka Medical, Ltd.,Tokyo, Japan)	Free-hand RTE	Skin and subcutaneous tissue strains before and after MLD to treat lymphedema.	None	Assessment	Thighs: The skin and subcutaneous tissue strains in lymphedema legs were significantly lower than those in normal legs. Calves: No significance was found, although the tissue strains in lymphedema legs tended to be lower than the normal legs. Correlations between pre-MLD and the MLD-induced changes in thigh and calf skin strains were significantly negative, but no correlation was found in subcutaneous tissue strains for lymphedema patients.
		35 healthy patients (70 legs)	Median (range): 37 (25-55)	-						
Suehiro et al [10]	2015	32 patients (62 legs with secondary lymphedema)	Median (range): 68 (37-89)	Stage 0 (n=16), Stage I (N=5), Stage II (n=35), Stage III (n=6)	Ultrasound elastography	Free-hand RTE	Skin and subcutaneous tissue strains	None	Assessment, staging	Thighs: No significant differences in skin or subcutaneous strains among all the lymphedema stages. Calves: Subcutaneous tissue strain for LDS was significantly lower than stages 0, II and late II lymphedema. Also, a significant decrease in skin strain in stage III compared with stages I and II were found for lymphedema. Finally, the skin strain for LDS was significantly lower than stage 0, I, II and late II lymphedema.
		10 patients (10 legs with LDS)	Median (range): 69 (53-79)	-						
Hayashi et al [8]	2015	18 patients with secondary lower limb lymphedema.	Mean (range): 52.9 (37-70)	Stage 0 (15 legs), stage I (3 legs), stage II (18 legs)	Ultrasound system (HI VISION Preirus, Hitachi Aloka Medical, Ltd.,Tokyo, Japan)	Elastography with the linear 18-5 MHz transducer in B mode (Hitachi Medical Corporation, Tokyo, Japan)	Area of red region in the subcutaneous tissue using the Image J software (National Institute of Health, Bethesda, MD, USA)	ICG lymphography	Assessment	The red region area (fluid content) measured in the three points of the affected legs had a correlation with the ICG lymphography. They were likely to increase as the ICG pattern progressed due to the aggravation of the disease.
		10 healthy patients.	Mean (range): 31.6 (24-52)	-						
Suehiro et al [11]	2014	15 patients with unilateral lower-extremity stage 2 secondary lymphedema	Median (range): 70 (37-87)	Stage II (n=15)	Ultrasound elastography	Free-hand RTE	Strains of the skin and subcutaneous tissue at the middle of the inner thigh and calf	None	Assessment	No significant differences were found between the affected and unaffected limbs for strains of the skin and subcutaneous tissue at any part of the leg. For the inner thigh, subcutaneous strain was higher in healthy patients compared to the patients with lymphedema.
		35 healthy patients	Median (range): 37 (25-55)	-						

Discussion

Lymphoscintigraphy is the procedure of choice when assessing lymphedema. However, it is costly, time-consuming, and invasive due to requiring an additional intradermal injection of a radionuclide [13]. Imaging tests of high-resolution like computed tomography (CT) and magnetic resonance imaging (MRI) have been proposed to be useful in the assessment and diagnosis of lower extremity lymphedema [14, 15]. However, these tests are expensive and expose patients to radiation. On the other hand, bioimpedance spectroscopy, tonometry, water displacement, perometry and circumferential tape measurements are noninvasive methods that evaluate lower extremity lymphedema; however, they are not able to identify structural skin and subcutaneous changes present in lymphedema. For that reason, UE is considered a novel noninvasive tool that can be potentially used in different lymphedema health centers at low-cost.

Our systematic review described the assessment of patients with LEL through the use of UE. Elastography is an objective quantitative tool that assesses the tissue elasticity and might estimate indirectly the fluid accumulation in lymphedema through the modification of the subcutaneous tissue elasticity [16]. For lymphedema patients, evaluation of this disease is based on the premise that subcutaneous tissue fibrosis will result in hardening of tissues. The two UE methods studied for LEL assessment were free-hand RTE and UE with transducer in B mode.

Free-hand RTE detects stiffness and hardness of tissues through the visualization of relative tissue displacement (strain distribution) in soft tissues [17] and has been used to differentiate malignant from benign tumors, with the idea that softer tissues are easier to deform under compression than harder tissues [18,19]. RTE has also been used to test neck, prostate, breast, and thyroid elasticity [20,21]. During RTE, a free-hand compression is applied with the probe being moved in a slight up-and-down motion over the area to be measured; the tissue elasticity is overlaid in real-time directly on the B image, where color scale ranges from red (soft) to blue (hard) [21]. In the RTE studies, skin and subcutaneous tissue strains were used as imaging biomarkers. The main concept of this method in lymphedema is that skin and subcutaneous tissue fibrosis progresses as lymphedema does, and as a result, it is assumed that these affected tissues are stiffer which means having a lower strain due to less deformity than tissue in non-affected lower extremities. Of all the studies regarding the use of RTE, only Suehiro et al did not find a significant difference in strains between the affected and non-affected legs, independent of the region [11]. An explanation for this result is that UE is not able to differentiate between water (high strain) and fibrosis (low strain) that might be present at the same time in lymphedema patients, and therefore, the different strains may overlap [22]. The authors explained that another cause may be due to the fact that enrolled patients were on stage II of lymphedema, and earlier stages of disease may have similar strains to normal tissues. However, they identified a higher subcutaneous strain in the normal legs of healthy people than in the affected legs in the inner thigh region. Given these results, a year later, Suehiro et al decided to extend their RTE study for assessment and staging of lymphedema patients [10]. They found different results by region evaluated in the leg in patients with different stages of lymphedema and lipodermatosclerosis. In the thigh, they revealed significantly lower strains in lipodermatosclerosis compared to stage 0 and II lymphedema. In addition, there was a significant decrease in skin calf strain in stage III compared with stages I and II. However, no difference was found between stage I or II and stage 0. The authors suggested these findings may be due to nonuniform inflammatory changes in the different regions of the affected leg and the heterogeneity of the patients included in the study, in addition to the presence of water in earlier stages that would also impact the results [23]. UE cannot measure the mechanical deformation of a specific tissue without being affected by the surrounding tissue. The presence of water in early stages of lymphedema may be shifted horizontally when compression is applied and, as a consequence, the strain measurements could potentially vary. In their last study in 2016, Suehiro et al applied the RTE method to measure skin and subcutaneous tissue strains before and after manual lymphatic drainage, which is a physical therapy treatment for lymphedema [9]. Interestingly, they reported skin and subcutaneous strain differences for each location, showing higher strains in the inner thigh and lower in the calf. Furthermore, skin and subcutaneous tissue strains in affected thighs and calves were lower than in normal thighs and calves, respectively, although the difference was significant only for strains in thighs [9]. Significant linear correlations were found for thigh and calf skin strain changes pre- to post-lymphatic drainage, but they did not find a significant difference for subcutaneous strains. This finding means that manual lymphatic drainage softens the skin and improves the strain. However, the absence of difference in subcutaneous tissue suggests a limitation in the assessment of deeper tissues. 

UE with transducer in B mode is a method based on the quasi-static method, where compression is applied to the tissue and an image of the strain produced is extracted from the difference between the reference image and the compressed image [24]. The calculation of this displacement is made by two-dimensional correlation of conventional ultrasound images (B-mode images) and may qualitatively show stiffness of tissue in a color image. Hayashi et al evaluated the use of this method in LEL and compared it with indocyanine green (ICG) lymphography patterns (linear, splash, stardust, diffuse) by measuring at three different points on the leg, including medial thigh, medial leg, and anterior ankle [8]. Blue color was displayed for hard tissue (eg, bone), green for soft tissue, yellow for softer tissue (eg, fat), and red for fluids. They found a moderately positive correlation between ICG pattern and the red areas, as well as between ICG pattern and the severity of disease at all three points of measure. However, no significant differences were found between the mean values of the red area and the stardust and diffuse patterns of ICG, probably due to development of fibrosis. The authors concluded that, while UE with transducer in B mode may be helpful in the assessment of moderate-to-severe stages of LEL, it would not be able to detect earlier stages of disease [8].

UE has studied other conditions such as skin tumors, subcutaneous T-cell lymphoma, mixed tumor of the scalp, systemic sclerosis, angiomatoid fibrous histiocytoma, skin or subcutaneous abscess, and post-irradiation neck fibrosis, however, these studies have been purely descriptive without any well-established UE parameter to differentiate between these conditions and lymphedema [25]. On the other hand, differentiation of LEL from other similar edematous conditions have been assessed previously using ultrasonography. High-resolution cutaneous ultrasonography was able to quantify dermal edema thickness and differentiate lymphedema from lipedema [26]. Lymphedema showed a dermal hypoechogenicity compared to lipedema that had a dermal echogenicity similar to normal skin. In contrast, lymphedema could not be differentiated from other entities when evaluating other parameters. For instance, lymphedema patients were found to have an increased subcutaneous echogenicity [23], which can also be found in inflammatory conditions like cellulitis. Another important condition that may overlap the lymphedema subcutaneous echogenicity is obesity, which can produce the same changes of lymphedema due to the blockage of the lymph fluid by fat that could also cause an increased subcutaneous echogenicity [23]. However, the use of UE in edematous diseases that resemble LEL has not been studied before and further studies are needed in this regard. Therefore, UE has limitations to differentiate the presence of other conditions that produce lower limb edema like obesity, cardiac or renal diseases or lower limbs venous insufficiency from the lymphedema changes. Differentiation of lymphedema with these other conditions cannot be assessed until the parameters and different strains for each condition, and the quantification of the presence of water in the skin and subcutaneous tissue that will allow the use of UE in early stages of lymphedema are established.

Strengths and Limitations

Our systematic review describes all articles in the English-language literature to date evaluating the use of UE for assessment and staging LEL, including UE methodology, imaging parameters, and results of these studies. Our work is limited by the heterogeneity of the populations studied in the included articles, in addition to the search, selection, and publication biases inherent to systematic reviews. However, our review is entirely descriptive, in alignment with the study aim.

## Conclusions

To our knowledge, our review is the first to describe the current literature on use of UE for assessment of patients with LEL. We concluded that UE is of benefit for patients in moderate-to-advance stages of disease. However, more effective methods are needed for evaluation of earlier stages. Further studies are needed for the assessment and establishment of parameters and cutoffs that determine staging and improvement of disease. In addition, incorporation of other imaging tools that detect flow distribution of lymphatic fluid may benefit these patients.
